# Proteomes of *Lactobacillus delbrueckii* subsp. *bulgaricus* LBB.B5 Incubated in Milk at Optimal and Low Temperatures

**DOI:** 10.1128/mSystems.00027-17

**Published:** 2017-09-19

**Authors:** Xiaochen Yin, Michelle R. Salemi, Brett S. Phinney, Velitchka Gotcheva, Angel Angelov, Maria L. Marco

**Affiliations:** a Department of Food Science and Technology, University of California, Davis, California, USA; b Proteomics Core Facility, UC Davis Genome Center, University of California, Davis, California, USA; c Department of Biotechnology, University of Food Technologies, Plovdiv, Bulgaria; University of Ghent

**Keywords:** *Lactobacillus*, fermentation, mammalian gut, proteomics

## Abstract

We identified the proteins synthesized by *Lactobacillus delbrueckii* subsp.* bulgaricus* strain LBB.B5 in laboratory culture medium (MRS) at 37°C and milk at 37 and 4°C. Cell-associated proteins were measured by gel-free, shotgun proteomics using high-performance liquid chromatography coupled with tandem mass spectrophotometry. A total of 635 proteins were recovered from all cultures, among which 72 proteins were milk associated (unique or significantly more abundant in milk). LBB.B5 responded to milk by increasing the production of proteins required for purine biosynthesis, carbohydrate metabolism (LacZ and ManM), energy metabolism (TpiA, PgK, Eno, SdhA, and GapN), amino acid synthesis (MetE, CysK, LBU0412, and AspC) and transport (GlnM and GlnP), and stress response (Trx, MsrA, MecA, and SmpB). The requirement for purines was confirmed by the significantly improved cell yields of *L. delbrueckii* subsp. *bulgaricus* when incubated in milk supplemented with adenine and guanine. The *L. delbrueckii* subsp. *bulgaricus*-expressed proteome in milk changed upon incubation at 4°C for 5 days and included increased levels of 17 proteins, several of which confer functions in stress tolerance (AddB, UvrC, RecA, and DnaJ). However, even with the activation of stress responses in either milk or MRS, *L. delbrueckii* subsp. *bulgaricus* did not survive passage through the murine digestive tract. These findings inform efforts to understand how *L. delbrueckii* subsp. *bulgaricus* is adapted to the dairy environment and its implications for its health-benefiting properties in the human digestive tract.

**IMPORTANCE** *Lactobacillus delbrueckii* subsp. *bulgaricus* has a long history of use in yogurt production. Although commonly cocultured with *Streptococcus salivarius* subsp. *thermophilus* in milk, fundamental knowledge of the adaptive responses of *L. delbrueckii* subsp. *bulgaricus* to the dairy environment and the consequences of those responses on the use of *L. delbrueckii* subsp. *bulgaricus* as a probiotic remain to be elucidated. In this study, we identified proteins of *L. delbrueckii* subsp. *bulgaricus* LBB.B5 that are synthesized in higher quantities in milk at growth-conducive and non-growth-conductive (refrigeration) temperatures compared to laboratory culture medium and further examined whether those *L. delbrueckii* subsp. *bulgaricus* cultures were affected differently in their capacity to survive transit through the murine digestive tract. This work provides novel insight into how a major, food-adapted microbe responds to its primary habitat. Such knowledge can be applied to improve starter culture and yogurt production and to elucidate matrix effects on probiotic performance.

## Abstract (precis)

*Lactobacillus delbrueckii* subsp. *bulgaricus* has a long history of use in yogurt production. Although commonly cocultured with *Streptococcus salivarius* subsp. *thermophilus* in milk, fundamental knowledge of the adaptive responses of *L. delbrueckii* subsp. *bulgaricus* to the dairy environment and the consequences of those responses on the use of *L. delbrueckii* subsp. *bulgaricus* as a probiotic remain to be elucidated. In this study, we identified proteins of *L. delbrueckii* subsp. *bulgaricus* LBB.B5 that are produced in higher quantities in milk at growth-conducive and non-growth-conductive (refrigeration) temperatures compared to laboratory culture medium and further examined whether those *L. delbrueckii* subsp. *bulgaricus* cultures were affected differently in their capacity to survive transit through the murine digestive tract. This work provides novel insight into how a major, food-adapted microbe responds to its primary habitat. Such knowledge can be applied to improve starter culture and yogurt production and to elucidate matrix effects on probiotic performance.

## INTRODUCTION

*Lactobacillus delbrueckii* subsp. *bulgaricus* is a member of the lactic acid bacteria (LAB), a diverse group of bacteria in the *Firmicutes* phylum named for the synthesis of lactic acid during fermentative growth. *L. delbrueckii* subsp. *bulgaricus* is particularly recognized for its importance in the production of yogurt, a fermented dairy product that originated centuries ago (1). During yogurt fermentation, protocooperation occurs between *L. delbrueckii* subsp. *bulgaricus* and *Streptococcus salivarius* subsp. *thermophilus*. Proteolytic *L. delbrueckii* subsp. *bulgaricus* hydrolyzes the milk casein into peptides satisfying the amino acid requirements of both species, while *S. salivarius* subsp. *thermophilus* provides *L. delbrueckii* subsp. *bulgaricus* with other metabolites, including formic acid, pyruvic acid, folic acid, fatty acids, and carbon dioxide (2–4).

Yogurt is increasingly recognized as a food that promotes human health. Epidemiological studies have shown positive associations between yogurt consumption and reduced risk for type 2 diabetes and cardiovascular diseases (5–7) and prevention of atopic dermatitis (8). The benefits of dairy have been largely limited to fermented as opposed to other dairy products (9). The reasons that fermented dairy products such as yogurt are supportive of human health are not known but might involve bacterial hydrolysis of milk proteins to bioactive peptides, synthesis of conjugated linoleic acids, or by probiotic effects on the human intestinal microbiota or epithelium (10). In that regard, the contributions of *L. delbrueckii* subsp. *bulgaricus* and *S. salivarius* subsp. *thermophilus* to improving lactose tolerance are already well established (11). It has also been suggested that *L. delbrueckii* subsp. *bulgaricus* could assist in the amelioration of acute diarrheal disorders (12) as well as enhance host systemic immunity, especially in elderly people (13–15).

As one of the most traditional dairy fermentation starters, *L. delbrueckii* subsp. *bulgaricus* has undergone adaptive evolution in milk to result in reduced genome sizes with specialized functions for growth in the nutrient-rich, milk environment (16, 17). However, identification of the precise and specialized adaptations of *L. delbrueckii* subsp. *bulgaricus* to milk have been limited to measuring its transcriptome when grown in whey (18) or in reconstituted skim milk together with *S. salivarius* subsp. *thermophilus* (4). Although one proteomics study identified proteins made by *L. delbrueckii* subsp. *bulgaricus* upon initial exposure to milk (19), only a few proteins were identified, and the totality of *L. delbrueckii* subsp. *bulgaricus* responses to the milk matrix were not examined. Thus, a systematic understanding of the functional adaptations of *L. delbrueckii* subsp. *bulgaricus* in milk is important to ultimately increase starter culture production efficiency and to elucidate the probiotic attributes of this species in the gastrointestinal (GI) tract.

Identification of *L. delbrueckii* subsp. *bulgaricus* responses to the dairy environment should also take into account low-temperature exposures. Fermented dairy products such as yogurts are typically preserved at refrigeration temperatures (4 to 7°C) prior to human consumption. Although such temperatures are not conducive for growth, *Lactobacillus* can remain metabolically active and adapt for survival under those conditions (20, 21). This was also recently shown for *Lactobacillus casei*, whereby certain proteins were synthesized exclusively or in greater quantities at low temperatures (22). Other proteins synthesized by *L. casei* in milk at 4°C were distinct from those produced under similar conditions in standard laboratory culture medium (22). Such cellular responses can have consequences for bacterial survival and persistence in the mammalian GI tract (23, 24). *L. casei* BL23 not only survived in larger quantities in the intestine when consumed in milk, but also was more efficacious in preventing intestinal inflammation in a mouse model of inflammatory bowel disease (25).

To investigate how *L. delbrueckii* subsp. *bulgaricus* adapts for growth and survival in milk, we employed shotgun, gel-free proteomics to identify the global cellular responses of *L. delbrueckii* subsp. *bulgaricus* LBB.B5 to milk under growth-conducive (37°C) and low-temperature (4°C) conditions and measured the effects of various incubation conditions on cell survival in the murine intestine.

## RESULTS

### Growth of *L. delbrueckii* subsp. *bulgaricus* LBB.B5-R in milk.

**FIG 1:**
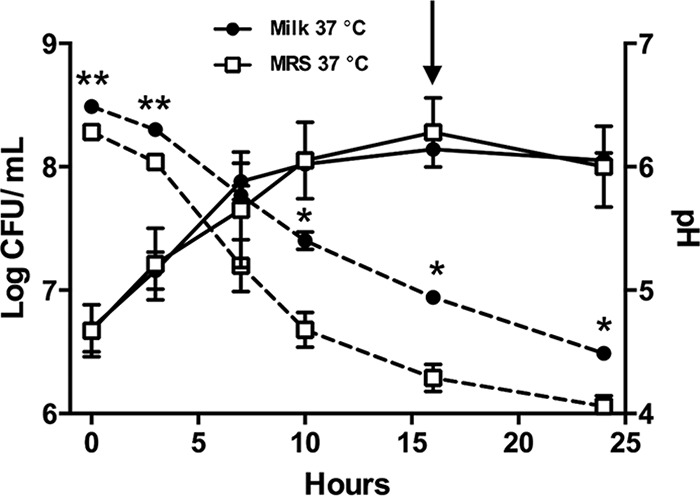
*L. delbrueckii* subsp. *bulgaricus* LBB.B5-R growth dynamics in MRS and milk. *L. delbrueckii* subsp. *bulgaricus* LBB.B5-R was incubated in either MRS or milk at 37°C for 24 h, and the cultures were sampled every 2 or 3 h for pH measurement and cell enumeration. Data are shown as mean ± standard error (SE). *, *P* < 0.05, and **, *P* < 0.01, by Student’s *t* test. The arrow indicates when the protein extraction was performed for the 37°C MRS and milk cultures.

*L. delbrueckii* subsp. *bulgaricus* LBB.B5-R grew similarly in milk and MRS and reached the same cell numbers (approximately 10^8^ CFU/ml) within 24 h (Fig. 1). Consistent with fermentative growth, the medium pH declined over time for all cultures (Fig. 1). Reductions in pH of MRS containing LBB.B5-R were significantly greater than that of milk (*P* < 0.05, Student’s *t* test). This result was possibly due to a better buffering capacity of the milk matrix (26). Transfer of the LBB.B5-R milk cultures from 37°C to 4°C and incubation at that low temperature for another 5 days resulted in no observable change in cell numbers (8.14 ± 0.02 log CFU/ml at 37°C and 8.08 ± 0.07 log CFU/ml at 4°C; *P* = 0.49) or milk pH (4.94 ± 0.04 log CFU/ml at 37°C and 4.91 ± 0.09 log CFU/ml at 4°C; *P* = 0.64).

### *L. delbrueckii* subsp. *bulgaricus* LBB.B5-R core-expressed proteins.

Proteins were extracted from *L. delbrueckii* subsp. *bulgaricus* LBB.B5-R cells incubated in MRS (*n =* 3) or milk (*n =* 3) for 16 h at 37°C (Fig. 1). Proteins were also collected from cells grown in milk for 16 h at 37°C and then transferred to 4°C for a subsequent 5-day incubation (*n =* 3). An average of 819 proteins were detected for each replicate culture, constituting 50% of the total 1,638 predicted proteins and encompassing all 20 categories from the Clusters of Orthologous Groups (COG) database represented in the genome of *L. delbrueckii* subsp. *bulgaricus* 2038 (see Table S1 in the supplemental material). Proteins for the complete glycolytic pathway were found in each of the expressed proteomes, thereby indicating that protein detection was sufficiently complete from each culture for metabolic reconstructions (see Table S2 in the supplemental material).

10.1128/mSystems.00027-17.1 TABLE S1  List of identified *L. delbrueckii* subsp. *bulgaricus* LBB.B5 proteins in this study. Download TABLE S1, XLSX file, 0.1 MB. Copyright © 2017 Yin et al. 2017 Yin et al. This content is distributed under the terms of the Creative Commons Attribution 4.0 International license.

10.1128/mSystems.00027-17.2 TABLE S2  Identified proteins involved in glycolysis. Download TABLE S2, XLSX file, 0.1 MB. Copyright © 2017 Yin et al. 2017 Yin et al. This content is distributed under the terms of the Creative Commons Attribution 4.0 International license.

**FIG 2:**
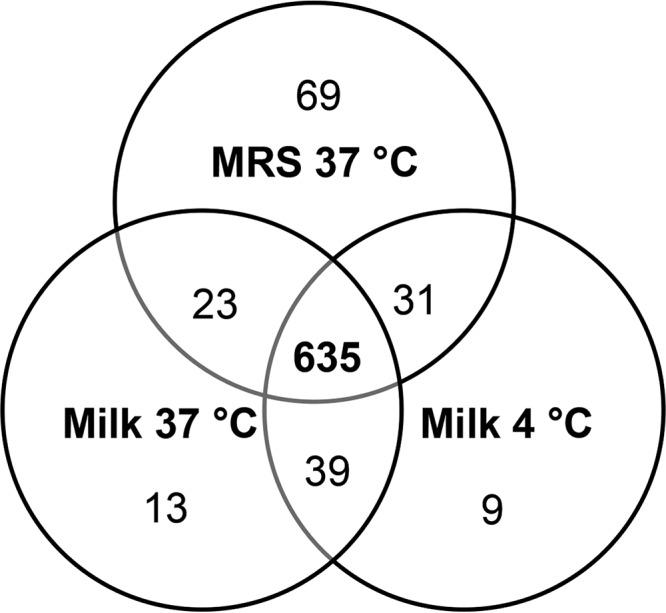
Shared and unique proteins produced by *L. delbrueckii* subsp. *bulgaricus* LBB.B5-R under different incubation conditions.

Despite the different media (MRS and milk) and incubation conditions (4 and 37°C), 635 proteins were repeatedly detected in all cultures (Fig. 2; Table S1). The majority of those core-expressed proteins belong to the COGs for “translation, including ribosome structure and biogenesis (J)” (113 proteins), “amino acid metabolism and transport (E)” (56 proteins), “nucleotide metabolism and transport (F)” (45 proteins), “carbohydrate metabolism and transport (G)” (43 proteins), and “cell wall structure and biogenesis and outer membrane (M)” (40 proteins). Notably, the biosynthesis pathway for 5-phosphoribosyl-1-pyrophosphate (PRPP), the precursor for nucleotide synthesis, was complete (6 proteins), as well as the aspartate (2 proteins) and threonine (5 proteins) biosynthetic pathways.

### Milk-associated proteins of *L. delbrueckii* subsp. *bulgaricus* LBB.B5-R.

**FIG 3:**
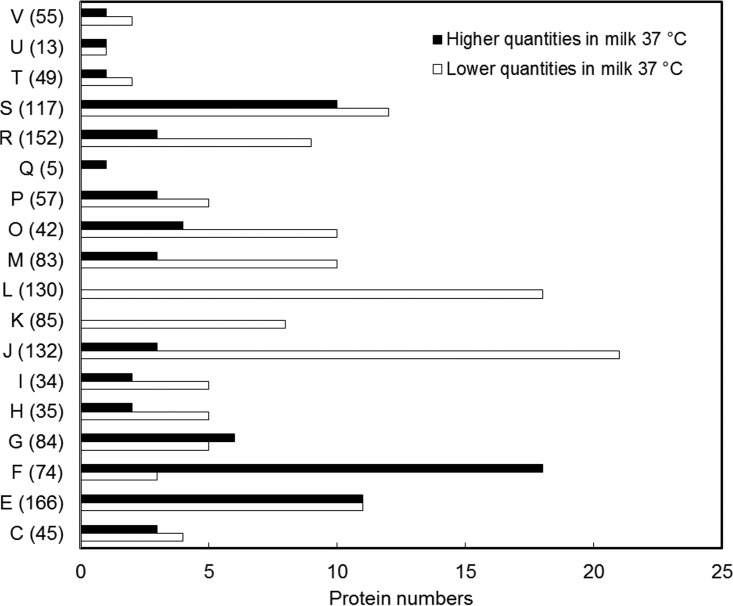
Milk-associated proteomes. Quantities of the significantly changed proteins are shown in their corresponding COG categories, and the number after each letter indicates total protein numbers within each COG (16). The functional categories are abbreviated as follows: C, energy production and conversion; E, amino acid metabolism and transport; F, nucleotide metabolism and transport; G, carbohydrate metabolism and transport; H, coenzyme metabolism; I, lipid metabolism; J, translation, including ribosome structure and biogenesis; K, transcription; L, replication, recombination, and repair; M, cell wall structure and biogenesis and outer membrane; O, molecular chaperones and related functions; P, inorganic ion transport and metabolism; Q, secondary metabolite biosynthesis, transport, and catabolism; R, general function predicted only; S, no functional prediction; T, signal transduction; U, intracellular trafficking, secretion, and vesicular transport; V, defense mechanisms.

A total of 203 *L. delbrueckii* subsp. *bulgaricus* LBB.B5-R proteins were recovered in significantly different quantities upon incubation in milk or MRS at 37°C. Among those proteins, 72 were either more abundant (37 proteins) or uniquely produced (35 proteins) after incubation in milk and included a high representation of the following COGs: “carbohydrate metabolism and transport (G)” (6 proteins [7.14% of total proteins in this COG]), “amino acid metabolism and transport (E)” (11 proteins [6.63%]), and “nucleotide metabolism and transport (F)” (18 proteins [24.32%]) (Fig. 3; see Table S3 in the supplemental material). Conversely, proteins involved in “replication, recombination, and repair (L)” (18 proteins [13.85%]), “transcription (K)” (8 proteins [9.41%]), and “translation (J)” (21 proteins [15.91%]) were significantly enriched during the incubation in MRS (a total of 131 proteins) (Table S3).

10.1128/mSystems.00027-17.3 TABLE S3  *L. delbrueckii* subsp. *bulgaricus* LBB.B5 proteins found in significantly different quantities in milk at 37°C incubation compared to MRS. Download TABLE S3, XLSX file, 0.1 MB. Copyright © 2017 Yin et al. 2017 Yin et al. This content is distributed under the terms of the Creative Commons Attribution 4.0 International license.

Among the proteins in the “carbohydrate metabolism and transport (G)” COG, β-galactosidase (LacZ) was found in the expressed proteomes in significantly larger quantities after *L. delbrueckii* subsp. *bulgaricus* growth in milk as opposed to glucose-containing MRS (Table S3). A mannose/glucose-specific phosphotransferase system (PTS) component II (ManM) was also uniquely detected in the milk cultures (Table S3). Because carbohydrate metabolism is important for energy generation in LAB, it was also notable that the glycolytic enzymes triosephosphate isomerase (TpiA), phosphoglycerate kinase (Pgk), and enolase (Eno) were more abundant upon incubation in milk than in MRS (Table S3). Other enzymes required for energy metabolism were also similarly increased, including a fumarate reductase flavoprotein (SdhA) and an NADP-dependent glyceraldehyde-3-phosphate dehydrogenase (GapN) (Table S3).

In the “amino acid metabolism and transport (E)” COG, quantities of methionine synthase (MetE), cysteine synthase (CysK), and two enzymes participating in aspartate synthesis, phosphoenolpyruvate carboxylase (LBU0412) and aspartate aminotransferase (AspC), were increased in LBB.B5-R in milk as opposed to MRS (Table S3). Amino acid transport proteins were also present in larger quantities, including proteins involved in glutamine uptake (GlnP [LBU1111] and GlnM [LBU0429]) (Table S3). In contrast, proteins required for proteolysis were elevated in MRS (PepA, PepC [LBU0224], PepC [LBU1473], and PepD) (Table S3).

*L. delbrueckii* subsp. *bulgaricus* incubation in MRS or milk also resulted in the production of different stress-responsive proteins. Thioredoxin (Trx), peptide methionine sulfoxide reductase (MsrA), a posttranscriptional regulator for genetic competence (MecA), and SsrA binding protein (SmpB) were each present in significantly larger amounts in *L. delbrueckii* subsp. *bulgaricus* after incubation in milk at 37°C. In MRS, a larger number of canonical, stress-responsive proteins were enriched, including chaperones (DnaK, DnaJ, and GrpE), proteases (ClpC, ClpE, and ClpX), two subunits of ATP synthase/ATPase (AtpD and AtpF), proteins involved in metal transport (CopA and CopB), and those required for DNA recombination and repair (RecA, RecN, MutL, MutS, and UvrC) (Table S3).

### Exogenous purine supplementation increases *L. delbrueckii* subsp. *bulgaricus* growth in milk.

**FIG 4:**
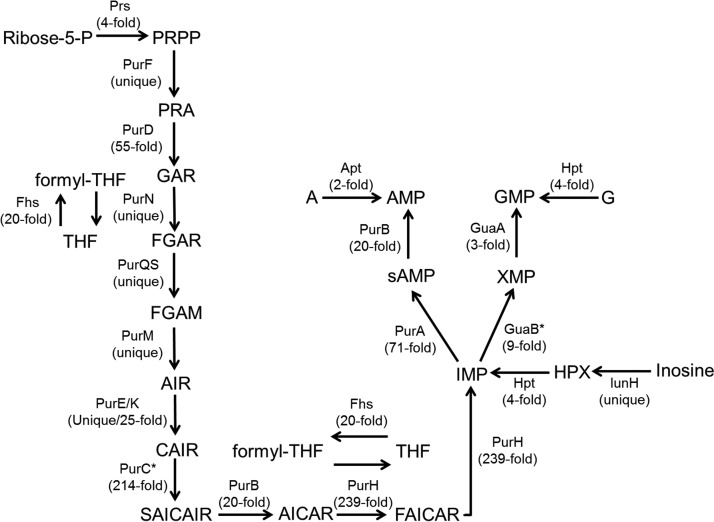
Significantly enriched proteins in purine *de novo* biosynthesis and salvage pathways in *L. delbrueckii* subsp. *bulgaricus* LBB.B5-R during milk incubation at 37°C. The symbol * indicates increased but not significant protein changes. The following abbreviations are used: ribose-5-P, ribose-5-phosphate; PRPP, phosphoribosyl pyrophosphate; PRA, phosphoribosyl amine; GAR, glycinamide ribonucleotide; (formyl-)THF, (formyl-)tetrahydrofolate; FGAR, formylglycinamide ribonucleotide; FGAM, formylglycinamidine ribonucleotide; AIR, aminoimidazole ribonucleotide; CAIR, phosphoribosyl carboxyaminoimidazole; SAICAIR, succinocarboxamide carboxyaminoimidazole ribonucleotide; AICAR, aminoimidazole carboxamide ribonucleotide; FAICAR, formaminoimidazole carboxamide ribonucleotide; sAMP, adenylsuccinate; A, adenine; G, guanine. Full names of the enzymes are listed as follows: Prs, PRPP synthase; PurF, PRPP amidotransferase; PurN, GAR transformylase; Fhs, formate-tetrahydrofolate ligase; PurQS, FGAM synthase; PurM, AIR synthase; PurEK, CAIR synthase; PurC, SAICAR synthase; PurB, adenylosuccinate lyase; PurH, bifunctional AICAR transformylase/IMP cyclohydrolase; PurA, adenylosuccinate synthase; Apt, adenine phosphoribosyltransferase; GuaB, IMP dehydrogenase; GuaA, GMP synthase; Hpt, hypoxanthine-guanine phosphoribosyltransferase; IunH, nucleoside (inosine/uridine) hydrolase.

Purine *de novo* biosynthesis starts with the conversion of PRPP to IMP, which is then modified to either AMP or GMP. *L. delbrueckii* subsp. *bulgaricus* enzymes catalyzing the conversion from PRPP to IMP were either uniquely produced (PurF, PurN, PurQ, PurS, PurM, and PurE) or significantly more abundant (PurD, PurK, PurB, PurH, and PurA) in milk (Fig. 4). Additionally, proteins required for the conversion of IMP to either AMP (PurA and PurB) or GMP (GuaA) were also found at greater levels in milk. The quantities of inosine-5′-monophosphate dehydrogenase (GuaB) were also larger, but the change was not significant (*P* = 0.06) (Fig. 4). Enzymes for the purine salvage and recycle pathways were also more abundant in milk, including nucleoside (inosine/uridine) hydrolase (IunH), adenine phosphoribosyltransferase (Apt), and hypoxanthine-guanine phosphoribosyltransferase (Hpt) (Fig. 4).

**FIG 5:**
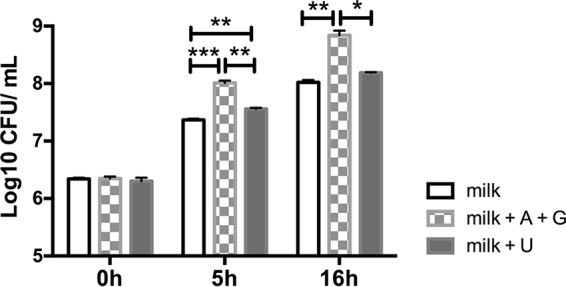
Increased cell numbers of *L. delbrueckii* subsp. *bulgaricus* LBB.B5-R during incubation in milk with the addition of purine (adenine [A] or guanine [G]) or pyrimidine (uracil [U]) bases. Data are shown as mean ± SE. ***, *P* < 0.001, **, *P* < 0.01, and *, *P* < 0.05, by Student’s *t* test.

To investigate the possibility that *L. delbrueckii* subsp. *bulgaricus* LBB.B5-R lacks access to nucleotides during growth in milk, exogenous purine bases (adenine and guanine) and pyrimidines (uracil) (20 μg/ml) were added to the ultrahigh-temperature (UHT)-treated milk, and *L. delbrueckii* subsp. *bulgaricus* growth was monitored. Adenine and guanine supplementation resulted in significantly larger quantities of *L. delbrueckii* subsp. *bulgaricus* after 5 and 16 h of incubation, respectively, compared to growth in milk alone (Fig. 5). The pH values of those cultures were also significantly reduced (*P* = 0.0001 after 5 h of incubation; *P* = 0.004 after 16 h of incubation). *L. delbrueckii* subsp. *bulgaricus* LBB.B5-R appears to be specifically limited in access to purines because the addition of uracil did not affect *L. delbrueckii* subsp. *bulgaricus* cell yields after 16 h of incubation (*P* = 0.07) (Fig. 5).

### Low-temperature-associated proteins of *L. delbrueckii* subsp. *bulgaricus* LBB.B5-R.

**FIG 6:**
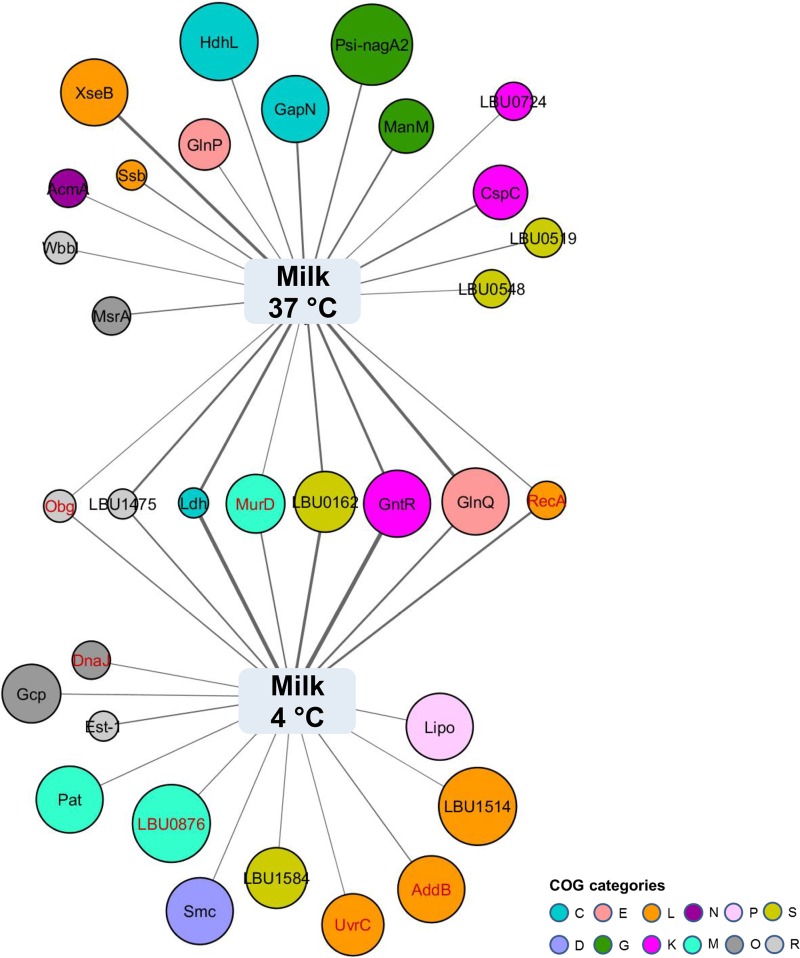
Low-temperature-associated proteomes. Proteins with *P* values of less than 0.1 and over 1.5-fold differences between the two conditions are shown. Node size negatively reflects the *P* value of the corresponding protein, and the edge width positively correlates to the protein NSAF value under each condition. Shared proteins produced in larger quantities in milk at 4°C incubation (versus milk at 37°C incubation) and MRS at 37°C incubation (versus milk at 37°C incubation) are colored in red. Each node color indicates a COG category. The functional categories are abbreviated as follows: C, energy production and conversion; D, cell cycle control and mitosis; E, amino acid metabolism and transport; G, carbohydrate metabolism and transport; L, replication, recombination, and repair; K, transcription; N, cell motility; M, cell wall structure and biogenesis and outer membrane; O, molecular chaperones and related functions; P, inorganic ion transport and metabolism; R, general function predicted only; S, no functional prediction.

A total of 33 proteins were present in significantly different quantities in milk after extended incubation at 4°C compared to 37°C (Fig. 6; see Table S4 in the supplemental material). Among those proteins, more than half (17) were either uniquely identified or enriched upon low-temperature incubation. Low-temperature-associated proteins included one transcriptional regulator (GntR), three proteins involved in “cell wall structure and biogenesis and outer membrane (M)” (LBU0876, LBU1584, and MurD), and one protein involved in metal ion transport (Lipo) (Fig. 6). Proteins involved in exopolysaccharide synthesis (EpsB and EpsC) were also specifically synthesized in milk at low temperature.

10.1128/mSystems.00027-17.4 TABLE S4  *L. delbrueckii* subsp. *bulgaricus* LBB.B5 proteins found in significantly different quantities in milk at 4°C compared to 37°C. Download TABLE S4, XLSX file, 0.1 MB. Copyright © 2017 Yin et al. 2017 Yin et al. This content is distributed under the terms of the Creative Commons Attribution 4.0 International license.

Other responses by *L. delbrueckii* subsp. *bulgaricus* to low-temperature incubation were directed at responding to environmental stress. DNA helicases (AddB and LBU1514) and proteins involved in DNA recombination and repair (UvrC and RecA) were increased in *L. delbrueckii* subsp. *bulgaricus* during incubation at 4°C (Fig. 6). The level of the chaperone protein DnaJ was also significantly higher after low-temperature incubation (Fig. 6). Notably, most of those proteins were enriched to even higher levels in cells incubated in MRS at 37°C (Table S4). Therefore, the environmental responses of *L. delbrueckii* subsp. *bulgaricus* in milk at 4°C were moderate compared to those of cells incubated in standard (MRS) laboratory culture medium.

### *L. delbrueckii* subsp. *bulgaricus* LBB.B5-R survival in the murine GI tract.

**FIG 7:**
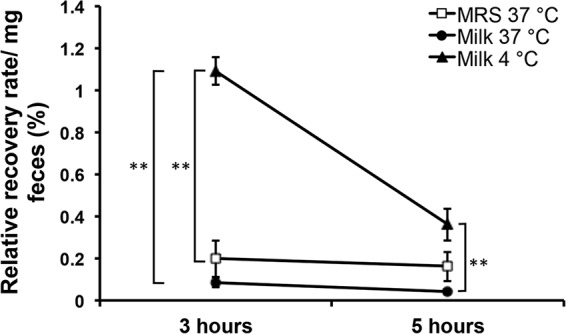
Low-temperature incubation transiently increased the survival of *L. delbrueckii* subsp. *bulgaricus* LBB.B5-R in the murine gut. Fecal samples were collected for *L. delbrueckii* subsp. *bulgaricus* enumeration 3 and 5 h after the feeding. The relative recovery rate was calculated by dividing the total number of cells fed to the mice by the cells recovered per milligram of fecal pellets. Data are shown as mean ± SE. **, *P* < 0.01 by Student’s *t* test.

To investigate the capacity of *L. delbrueckii* subsp. *bulgaricus* LBB.B5-R to survive passage through the digestive tract in different carrier matrices, BALB/c mice were fed *L. delbrueckii* subsp. *bulgaricus* LBB.B5-R suspensions that were incubated under the same conditions used for proteome analyses (MRS at 37°C for 16 h, milk at 37°C for 16 h, or cultured in milk and then maintained at 4°C for 5 days). The *L. delbrueckii* subsp. *bulgaricus* LBB.B5-R cell suspensions (ranging from 10^6^ to 10^7^ cells per feeding) were fed to each of the mice for 5 consecutive days. Remarkably, culturable LBB.B5-R cells were below the detection limit (3 CFU of the rifampin-resistant LBB.B5/mg feces) 24 h after each feeding. The carrier medium (milk or MRS) and incubation conditions yielded little notable difference, except there were significantly higher numbers of viable *L. delbrueckii* subsp. *bulgaricus* cells in the stools 3 and 5 h after the first feeding when the organism was provided in the cooled (4°C incubated) milk (Fig. 7).

## DISCUSSION

Fermented foods have been a staple of the human diet since the start of organized food production and development of agricultural practices. Yogurt is believed to have first appeared in ancient Turkey in the 8th century AD, although no accurate records of its origin are available (27). Genome investigations have strongly indicated that the continuous cultivation of *L. delbrueckii* subsp. *bulgaricus* in milk resulted in strains with genetic adaptations and genome-size reductions indicative of specialization to that specific habitat (17). However, by identifying and comparing the expressed proteomes of *L. delbrueckii* subsp. *bulgaricus* LBB.B5-R produced in milk during growth-conducive and non-growth-conducive (low-temperature) conditions, we found that the metabolic activities of *L. delbrueckii* subsp. *bulgaricus* remain dynamic and depend on the environmental context in which the organism is grown. The findings also demonstrate the limited capacity of this organism to persist in the mammalian intestine, even when consumed in the dairy matrix.

Milk is primarily composed of lactose, in addition to small quantities of glucose, fructose, and oligosaccharides (28). Therefore, the presence of increased levels of β-galactosidase (LacZ) in LBB.B5-R when grown in milk as opposed to glucose-containing MRS confirms that the identified expressed proteomes were sufficient to confirm the primary metabolic activities of this strain. Additionally, we also found that a glucose/mannose-specific PTS component (ManM) was enriched in the presence of milk. This result was consistent with higher quantities of ManM in *L. casei* Zhang when cultured in soymilk (29) and increased transcripts for the glucose/mannose-specific PTS during *L. delbrueckii* subsp. *bulgaricus* 2038 incubation in whey (18). Taken together, these findings indicate that *L. delbrueckii* subsp. *bulgaricus* LBB.B5-R consumes lactose and other sugars (such as glucose) during growth in (soy) milk or whey. Although the consequences on energy metabolism are not yet clear, it is notable that certain proteins in that pathway (e.g., Eno, Pgk, and TpiA) and other energy generation, redox-balancing enzymes (e.g., GapN) (17) were also enriched in milk.

All enzymes required for purine biosynthesis and recycling were produced in larger quantities by *L. delbrueckii* subsp. *bulgaricus* LBB.B5-R during growth in milk compared to MRS. The magnitude of these differences is evident with PurH, an enzyme essential for purine biosynthesis, which was produced by *L. delbrueckii* subsp. *bulgaricus* in >200-fold larger quantities in milk. The lack of sufficient purines in milk was confirmed by higher cell yields when *L. delbrueckii* subsp. *bulgaricus* LBB.B5-R was grown in the presence of exogenous adenine and guanine. The need for purine biosynthesis and salvage proteins in milk is supported by transcriptome analyses of *L. delbrueckii* subsp. *bulgaricus* 2038 and ATCC BAA-365 in whey and skim milk, respectively (4, 18). The production of purine biosynthetic proteins was also elevated in other LAB (e.g., *Lactobacillus helveticus*, *L. casei*, and *Lactococcus lactis*) when grown in milk (29–31). However, genes coding for purine metabolism were downregulated in *L. delbrueckii* subsp. *bulgaricus* when cocultured with *S. salivarius* subsp. *thermophilus*. This difference might have been due to *S. salivarius* subsp. *thermophilus* production of formic acid and folic acid, essential precursors for purine biosynthesis (4). Lastly, it is also notable that *L. delbrueckii* subsp. *bulgaricus* LBB.B5-R growth in milk was not increased when the pyrimidine uracil was added. This result might have been due to the high concentrations of orotate, a pyrimidine precursor, in milk (32). Correspondingly, quantities of orotate phosphoribosyltransferase (PyrE) were higher (*P* = 0.09) in milk than in MRS.

Amino acid metabolism was also changed in *L. delbrueckii* subsp. *bulgaricus* between the milk and MRS cultures. Although milk is a rich source of nitrogen (33), the production of aspartate, cysteine, and methionine biosynthetic enzymes was elevated in *L. delbrueckii* subsp. *bulgaricus* LBB.B5-R in milk compared to MRS. The need for cysteine and methionine was possibly due to the low abundance of those sulfur-containing amino acids in bovine milk (34). Also distinct from MRS was the increase in glutamine transport proteins (LBU1111 and LBU0429). Glutamine is required for nucleotide and amino acid synthesis (35), and its production by glutamine synthase was also enhanced in *L. lactis* NCDO763 when grown in skim milk compared to synthetic medium M17 (30). Remarkably, enzymes required for proteolysis and protein turnover were more abundant in MRS than in milk. This finding is notable because an important function of *L. delbrueckii* subsp. *bulgaricus* in yogurt fermentations is proteolysis and the release of peptides and amino acids to *S. salivarius* subsp. *thermophilus* (4). When cocultured with *S. salivarius* subsp. *thermophilus*, higher expression of the *L. delbrueckii* subsp. *bulgaricus* extracellular protease (PrtB) responsible for the first step in the proteolysis of caseins was found to satisfy the amino acid requirement for both microbes (4). However, in the absence of *S. salivarius* subsp. *thermophilus*, *L. delbrueckii* subsp. *bulgaricus* might then first consume available peptides and amino acids before initiating proteolysis.

Lastly, proteins required for adaptation to environmental stresses were differentially produced by *L. delbrueckii* subsp. *bulgaricus* in milk and MRS. Thioredoxin (Trx) and peptide methionine sulfoxide reductase (MsrA) were both detected in larger quantities in milk. Both of those proteins are required for responding to oxidative stress (36), and this finding is consistent with the responses of other LAB to the oxidative environment of milk (37). Conversely, in the presence of *S. salivarius* subsp. *thermophilus*, it might be expected that fewer stress-related proteins would be produced in *L. delbrueckii* subsp. *bulgaricus*, in part, because of the generation of a more supportive environment during *S. salivarius* subsp. *thermophilus* growth (4). Notably, however, the majority of other differentially expressed, stress-responsive proteins were found in larger quantities or uniquely produced by *L. delbrueckii* subsp. *bulgaricus* in MRS not milk. Those proteins, encompassing chaperones, Clp proteases, heavy metal transporters, and two subunits of the ATPase/ATP synthase, are required for LAB adaptations to acid conditions (38) and therefore consistent with the lower pH of the MRS as opposed to the milk cultures. These findings also provide new evidence for the mechanisms by which *L. delbrueckii* subsp. *bulgaricus* is able to tolerate acidic and oxidative environments.

Transfer of *L. delbrueckii* subsp. *bulgaricus* milk cultures from 37 to 4°C also resulted in the increased synthesis of RecA and other stress-response-related proteins. RecA synthesis was similarly increased in *L. casei* BL23 at 4°C (22), thereby indicating the importance of DNA recombination and repair for LAB at reduced temperatures. It is notable, however, that the effects of exposure of *L. delbrueckii* subsp. *bulgaricus* to 4°C in milk were still relatively minimal compared to the production levels of those proteins under more acidic conditions at 37°C in MRS. Moreover, only relatively few proteins were differentially abundant in milk at 4°C compared to 37°C incubation. Because there was no decline in *L. delbrueckii* subsp. *bulgaricus* cell numbers 5 days after transfer to 4°C, the detected expressed proteomes were likely the outcome of adaptive and sustained changes in cellular functions to the reduced temperature. Notable among those proteins produced at 4°C were enzymes for exopolysaccharide (EPS) synthesis. Increased production of EpsB and EpsC was observed with putative functions in EPS polymerization and chain length determination (39). This is of special interest because EPS contributes to the unique rheological property and texture of fermented milk, and moreover, these sugar polymers might promote the intestinal survival of the producing strain as well as exert certain health benefits (23).

The potential for carrier-matrix-induced effects on probiotic efficacy (23, 40) led us to also test the capacity of the *L. delbrueckii* subsp. *bulgaricus* cultures to survive transit through the murine GI tract. Irrespective of growth medium and incubation temperature, LBB.B5-R survived very poorly and was not reliably detected in the stools 24 h after consumption. Similarly, Marteau et al. found that *L. delbrueckii* subsp. *bulgaricus* strain LB9 survived in less than 1% of the initial cell numbers after passing the gastric compartment using an *in vitro* gastrointestinal (small intestine) model (41). This level of survival in the mouse digestive tract is lower than for other lactobacilli, including *L. plantarum*, *L. casei*, and *Lactobacillus acidophilus* (22, 41–43). However, even with the low levels of survival overall, ingestion of *L. delbrueckii* subsp. *bulgaricus* LBB.B5-R in cooled milk yielded at least an initial increase in viable cells in the mouse stools. These findings support the premise that there might be at least a transient benefit to consuming *L. delbrueckii* subsp. *bulgaricus* cultures prepared under similar conditions. Additional studies are needed to determine the impact of coculturing in the presence of *S. salivarius* subsp. *thermophilus* and the final yogurt carrier matrix on *L. delbrueckii* subsp. *bulgaricus* performance in the GI tract.

In conclusion, our approach to identify and compare the expressed proteomes of *L. delbrueckii* subsp. *bulgaricus* in milk and low-temperature conditions supports the elucidation of species and strain adaptations to dairy food matrices. Our results show the need to also consider other environmental factors, such as low-temperature storage and the delivery matrix, which can be used to inform fermentation optimization protocols and support probiosis in the mammalian intestine.

## MATERIALS AND METHODS

### Bacterial strains and culture conditions.

*L. delbrueckii* subsp. *bulgaricus* LBB.B5 was obtained from the LBB Culture Collection (LB Bulgaricum Plc, Sofia, Bulgaria). A spontaneous rifampin-resistant mutant of *L. delbrueckii* subsp. *bulgaricus* LBB.B5 (LBB.B5-R) was used for this study by selecting a single-colony isolate grown on deMan, Rogosa, and Sharpe agar (MRS) (BD, Franklin Lakes, NJ) containing 50 μg/ml rifampin (Thermo Fisher Scientific, Waltham, MA). When indicated, the strain was incubated in ultrahigh temperature (UHT)-processed 2% reduced fat milk (Gossner Foods, Logan, UT). To measure cell growth in response to exogenous nucleobases, purine (adenine and guanine) or pyrimidine (uracil) bases were added to the milk to a final concentration of 20 μg/ml. Bacterial cultures were serially diluted, plated onto MRS agar containing 50 μg/ml rifampin, and incubated at 37°C for 2 days prior to colony enumeration.

Equal quantities of *L. delbrueckii* subsp. *bulgaricus* LBB.B5-R (approximately 10^6^ CFU/ml) were inoculated into either MRS (*n =* 3) or milk (*n =* 6) and incubated at 37°C without aeration for 24 h. After 16 h at 37°C, three of the cultures in milk were transferred to 4°C and incubated for the subsequent 5 days. For all cultures, pH was periodically measured with the S20 SevenEasy (Mettler-Toledo LLC, Columbus, OH), and numbers of viable cells were estimated on MRS plates containing 50 μg/ml RIF.

### Proteome analysis.

*L. delbrueckii* subsp. *bulgaricus* LBB.B5-R cells were collected after 16 h of incubation in MRS or milk at 37°C and after an extended 5-day incubation at 4°C in milk. Total cellular proteins were extracted as previously described with minor modifications (22). Small amounts of 1 M sodium hydroxide were added to bacterial cultures in addition to 1 M trisodium citrate and buffered saline solution (pH 7.0) (0.145 M sodium chloride, 0.016 M sodium β-glycerophosphate, 0.1% Tween 80) to prevent casein precipitation during centrifugation (8,000 × *g* for 5 min at 4°C). Cell pellets were suspended in 50 mM ammonium bicarbonate buffer (pH 8.0) with 1 mM phenylmethylsulfonyl fluoride (PMSF) to inhibit protease activity. Soluble proteins were collected by centrifugation and stored at −20°C until further analysis.

The Bradford assay was used for protein quantification (Bio-Rad Protein assay kit II; Bio-Rad, Hercules, CA). A total of 50 μg protein extracted from each *L. delbrueckii* subsp. *bulgaricus* LBB.B5-R culture was subjected to in-solution reconstitution using 6 M urea followed by reducing and alkylating steps (22). Samples were digested in solution by Lys-C protease and trypsin and then subjected to bottom up proteomics by high-performance liquid chromatography (LC) coupled with tandem mass spectrophotometry (MS/MS) on a Thermo Scientific Q Exactive Orbitrap mass spectrometer in conjunction with a Proxeon Easy-nLC II high-performance liquid chromatograph (Thermo Scientific, Waltham, MA) and Proxeon nanospray source. Samples were processed and ran at the UC Davis Proteomics Core Facility (http://proteomics.ucdavis.edu/). Peptides were loaded onto a 100-µm by 25-mm Magic C_18_ 100-Å 5U reverse-phase trap, where they were desalted online before being separated using a 75-µm by 150-mm Magic C_18_ 200-Å 3U reverse-phase column. Peptides were eluted using a 180-min gradient with a flow rate of 300 nl/min. An MS survey scan was obtained for the *m*/*z* range 350 to 1,600. MS/MS spectra were acquired using a top 15 method, in which the top 15 ions in the MS spectra were subjected to high-energy collisional dissociation (HCD). An isolation mass window of 1.6 *m*/*z* was used for the precursor ion selection, and a normalized collision energy of 27% was used for fragmentation. A 5-s duration was used for the dynamic exclusion. Three technical replicates were run for each sample and combined for further data analysis.

### Data analysis.

Tandem mass spectra were extracted and the charge state deconvoluted by Proteome Discoverer (Thermo Scientific, San Jose, CA). All MS/MS samples were analyzed using X! Tandem (The GPM; http://www.thegpm.org/tandem [version TORNADO, 2013.02.01.1]). X! Tandem was set up to search *Lactobacillus delbrueckii* subsp. *bulgaricus* strain 2038 (version 20140416 [3,776 entries]) and the cRAP database of common laboratory contaminants (http://www.thegpm.org/crap [114 entries]) plus an equal number of reverse protein sequences assuming the digestion enzyme trypsin. X! Tandem was searched with a fragment ion mass tolerance of 20 ppm and a parent ion tolerance of 20 ppm. An iodoacetamide derivative of cysteine was specified in X! Tandem as a fixed modification. Deamidation of asparagine and glutamine, oxidation of methionine and tryptophan, sulfone of methionine, tryptophan oxidation to formylkynurenin of tryptophan, and acetylation of the N terminus were specified in X! Tandem as variable modifications. *L. delbrueckii* subsp. *bulgaricus* 2038 was selected for annotation because a greater proportion of LBB.B5-R peptide sequences could be matched to this strain (16). Annotated proteins were further classified to their corresponding Clusters of Orthologous Groups (COG).

Scaffold (version Scaffold_4.0; Proteome Software, Portland, OR) was used to validate MS/MS-based peptide and protein identifications with the parameters of 99% identity for protein probability and 95% for peptide probability, which resulted in 892 proteins at a 3% decoy false-discovery rate (FDR) and 747,782 spectra at a 0.06% decoy FDR according to previously described methods (44). Proteins were only considered to be present when identified in at least 2 out of 3 replicates. Normalized spectrum abundance factors (NSAF) were used for protein quantification. The Student’s *t* test was performed on the natural log-transformed NSAF values (where 0 was replaced with 0.0001 before transformation) followed by FDR adjustment in R studio (45). Network co-occurrence was visualized in Cytoscape (version 3.2.1) (46).

### Mouse study design.

All procedures were performed under the protocol approved by the UC Davis Animal Care and Use Committee (protocol no. 17899). Twenty-four female BALB/c mice (5 weeks old) (Harlan, Livermore, CA) were housed four per cage and given free access to food and water on a 12-h light/dark cycle. After acclimation for 5 days, the mice were randomly assigned to three groups (*n =* 8 per group) for administration of *L. delbrueckii* subsp. *bulgaricus* LBB.B5-R incubated in either MRS or milk at 37°C for 16 h or incubated in milk at 37°C for 16 h and then at 4°C for 5 days. A total volume of 50 µl *L. delbrueckii* subsp. *bulgaricus* LBB.B5-R was provided to the mice on the tip of a ball-tipped gavage needle. The mice were fed *L. delbrueckii* subsp. *bulgaricus* LBB.B5-R for 5 consecutive days in average quantities of 1.32 × 10^7^ (standard error [SE], 1.63 × 10^6^) cells incubated in MRS at 37°C for 16 h, 6.29 × 10^6^ (SE, 6.53 × 10^5^) cells incubated in milk at 37°C for 16 h, or 2.49 × 10^6^ (SE, 4.25 × 10^5^) cells incubated in milk and then maintained at 4°C for 5 days. Freshly expelled fecal pellets were collected at 0, 3, and 5 h after the first administration and then every 24 h for the remaining 4 days for *L. delbrueckii* subsp. *bulgaricus* enumeration. The relative recovery rate of *L. delbrueckii* subsp. *bulgaricus* was calculated following the equation relative recovery rate = (rifampin-resistant cells recovered per mg mouse fecal sample/total *L. delbrueckii* subsp. *bulgaricus* LBB.B5-R cells fed to mouse) × 100%.

### Availability of data.

All data have been deposited in the Massive proteomics repository (Massive ID MSV000080650) and submitted to Proteome Exchange (PXD006110) through the Massive repository.
